# Hand Pilomatrixoma: A Rare Localization

**DOI:** 10.7759/cureus.38492

**Published:** 2023-05-03

**Authors:** Nabila Sellal, Mohamed El Hfid

**Affiliations:** 1 Faculty of Medicine and Pharmacy of Tangier, Abdelmalek Essaadi University, Tangier, MAR; 2 Radiotherapy Department, University Hospital of Tangier, Tangier, MAR

**Keywords:** benign, adult, hand, calcified epithelioma of malherbe, pilomatrixoma

## Abstract

Pilomatrixoma, also known as calcified epithelioma of Malherbe, is the most common tumor of the hair follicle. It is a benign tumor in a young adult. Pilomatrixoma is most commonly located in the head and neck. It is usually misdiagnosed and confused with other skin lesions. The authors report a rare localization of this tumor in the left hand in a 40-year-old patient treated surgically without recurrence after four years of follow-up.

## Introduction

Pilomatricoma, or calcified epithelioma of Malherbe, is a type of benign, rare skin tumor that develops at the expense of the hair matrix and is the most common tumor of the hair follicle [1,2]. These tumors are most common in children and young adults, and they are often confused with other types of skin lesions. Pilomatricoma occurs most frequently in the head and neck, and localization in the limbs remains an exceptional occurrence [3]. Herein, the authors provide a case report of a pilomatricoma of the hand in a 40-year-old patient, where the rarity of the localization is the particularity of this case.

## Case presentation

**Figure 1:**
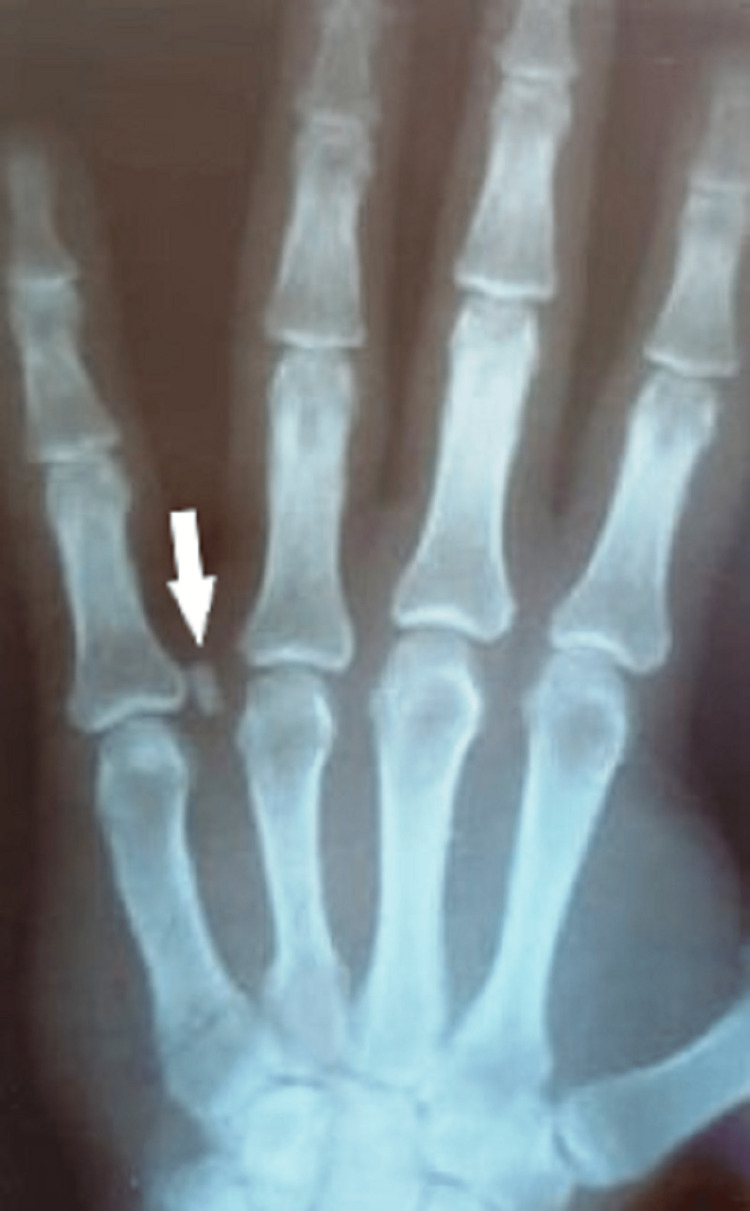
Left-hand X-ray showing well-limited soft tissue centimetric calcification.

We report a clinical case of a 40-year-old male patient with no past medical history who received a consultation for swelling on the palmar surface of the left hand, which demonstrated a progressive increase in size over three years. Six months before his consultation, the patient felt discomfort flexing his fingers, which impacted some of his manual activities. A clinical exam showed a nodular mass in the fifth finger measuring 1 cm in diameter that was hard, painless, and adherent to the skin. An X-ray of the left hand showed a well-limited centimetric calcification of the soft tissue (Figure 1).

**Figure 2:**
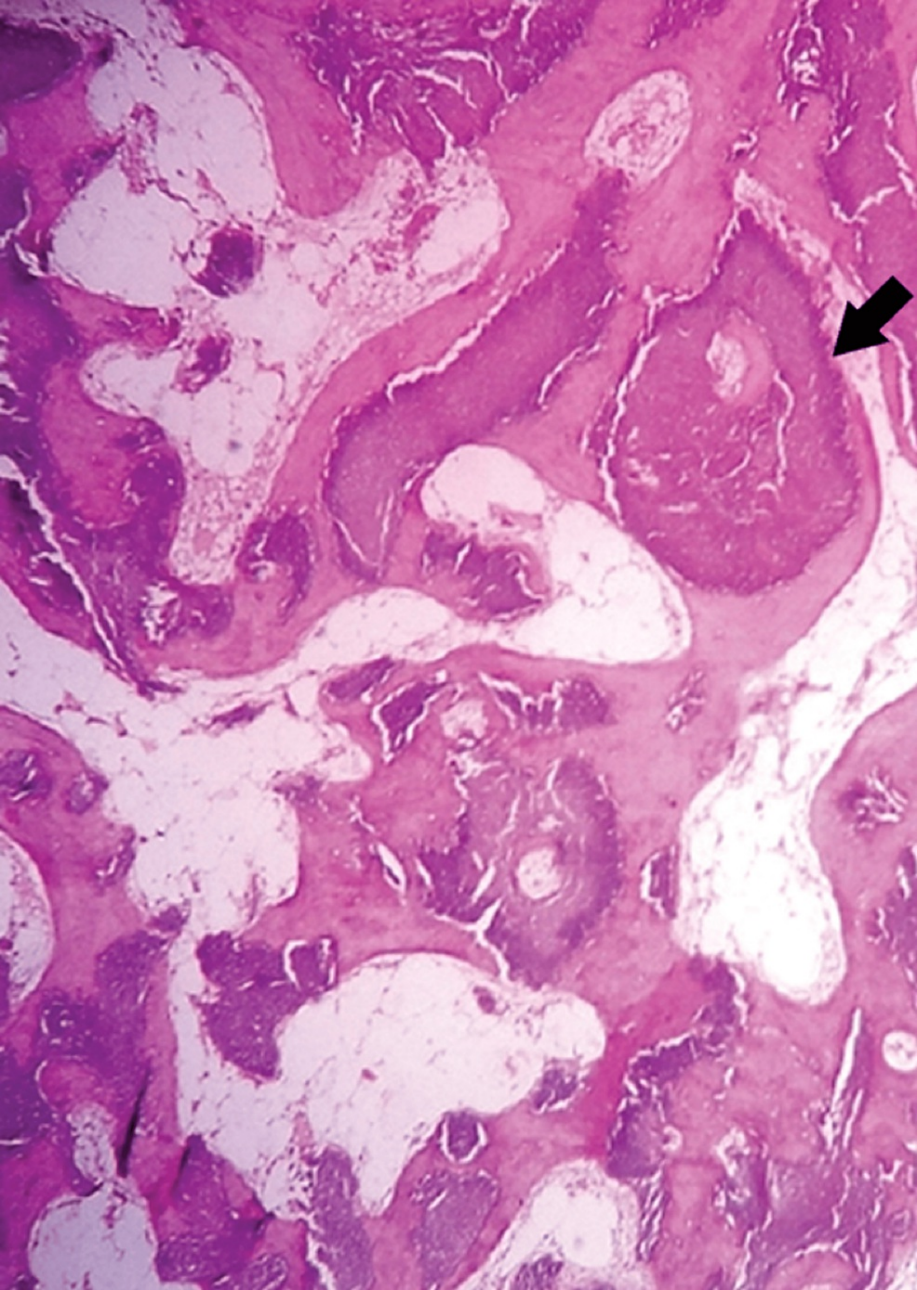
Tumor proliferation organized in lobules (20× magnification).

A large resection of the nodule was performed under local anesthesia. The histological study found a nodular mass measuring 1 cm × 0.5 cm × 0.5 cm with a smooth surface on macroscopy, which was brownish with a hard and ossified consistency. The microscopic study showed tumor proliferation organized in lobules and nests, bordered by basaloid cells, and containing regular adipocytes without cytonuclear atypia (Figure 2).

**Figure 3:**
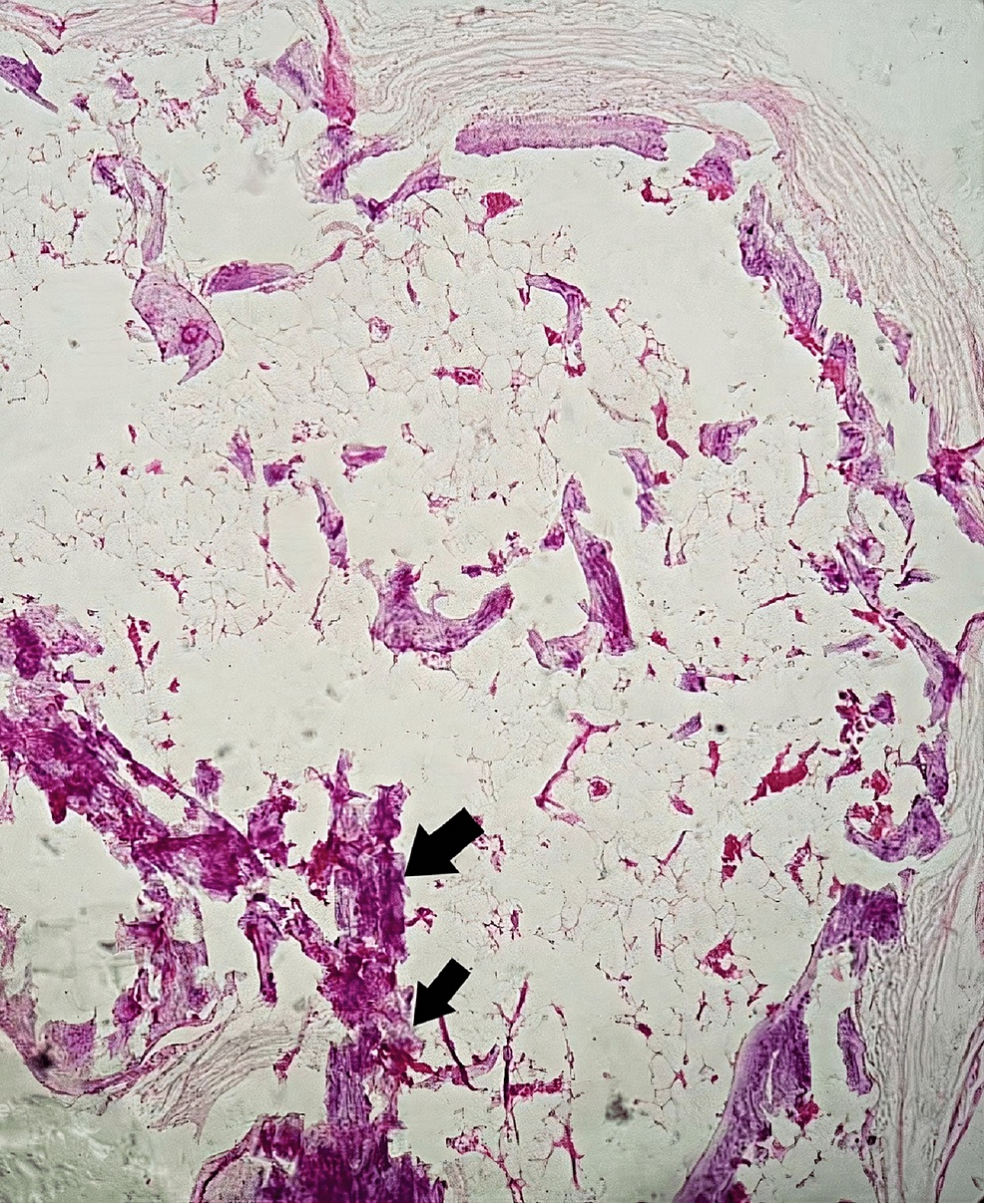
Presence of calcification and ossification (40× magnification).

Calcification and ossification phenomena were found without signs of malignancy (Figure 3).

Post-operative follow-up was normal, with complete physical recovery and functional integrity of the left hand. After four years of follow-up, the patient is still in complete remission.

## Discussion

Pilomatricoma, or Malherbe’s mummified epithelioma, is a benign hypodermic tumor developed from the matrix of the hair follicle. Described for the first time by Wickens in 1858, the development of its clinical and anatomopathological characteristics was performed by Malherbe and Chenantais in 1880, and then by Forbis and Helwig in 1961 [4-6].

Pilomatricoma is the most common tumor of the hair follicle but is often unrecognized, misdiagnosed, or confused with other skin lesions [1]. The prevalence and incidence of this tumor type are not known, but it occurs most often in young adults, usually under 20 years old, with a second peak in frequency between ages 50 and 65 [7-9]. Pilomatricoma is more common in women, with a female-to-male ratio of 1:5 [9]. In this case, the patient is 40 years old and male.

In a series of 346 cases of pilomatricoma, Pirouzmanesh et al. found that the most frequent locations were in the head and neck (70% of cases), followed by the upper limbs (15.3% of cases) [3]. Although a few cases of pilomatricoma of the upper limb have been reported in the literature, particularly in the arm and forearm [11-14], no case of metacarpal pilomatricoma has been reported in the literature.

Clinically, pilomatricoma typically presents as single or multiple subcutaneous nodules that are asymptomatic, round or oval, irregular, hard or firm in consistency, and adherent to the superficial plane. The skin next to the lesion is often bluish, but the skin looked normal in our case. Tumor calcification is present in 80% of cases, which explains the hard consistency of the nodule and the differential diagnosis with subcutaneous osteoma [15], as in our case. In the literature, pilomatricomas range in size from 0.1 to 6 cm, with an average size of 1.2 cm [3]. Giant pilomatricomas of the upper limb and parotid gland that are larger than 5 cm in size have been reported [13,16].

The use of paraclinical examinations is not mandatory; standard radiography is useful when there is a suspicion of pilomatricoma only when it is significantly calcified [15], as is our clinical case. Ultrasound can help in the diagnosis, especially when deep involvement is suspected, but the ultrasonographic aspect is often nonspecific; the existence of a posterior shadow cone indicates the presence of calcifications. Unfortunately, magnetic resonance imaging and computed tomography do not add any additional information.

The diagnostic confirmation of pilomatricoma is histological. The morphological appearance of pilomatricoma shows the presence of basophils and mummified cells in varying proportions depending on the age of the lesions, with calcifications and a giant cell inflammatory reaction [10].

Treatment for pilomatricoma consists of complete surgical excision, which is the main and gold-standard treatment to prevent recurrence [4]. The prognosis for pilomatricoma is generally good, with recovery without recurrence being typical after total surgical excision [8,14].

## Conclusions

Pilomatricoma is an often unrecognized benign tumor of the hair follicle that frequently affects the head and neck region. Hand localization in pilomatricoma remains exceptional; its diagnosis is clinical, its confirmation is histological, and its therapeutic management is surgical. The prognosis is favorable, but follow-up is mandatory to detect recurrence.
